# Management of type 2 diabetes and its prescription drug cost before and during the economic crisis in Greece: an observational study

**DOI:** 10.1186/1472-6823-14-23

**Published:** 2014-03-05

**Authors:** Stavros Liatis, Stavroula Papaoikonomou, Asimina Ganotopoulou, Athanasia Papazafiropoulou, Constantinos Dinos, Marios Michail, Apostolos Xilomenos, Andreas Melidonis, Stavros Pappas

**Affiliations:** 1 First Department of Propaedeutic Medicine, Diabetes Center, Athens University Medical School, Laiko Hospital, Ag. Thoma 17, 11527 Athens, Greece; 2 Diabetes Center, Nikaia Hospital, Piraeus, Greece; 3 Diabetes Center, Tzanio Hospital, Piraeus, Greece

**Keywords:** Type 2 diabetes, Prescription cost, Economic crisis, Cardiovascular risk

## Abstract

**Background:**

The aim of the present study is to examine the clinical indices related to cardiovascular risk management of Greek patients with type 2 diabetes, before and after the major economic crisis that emerged in the country.

**Methods:**

In this retrospective database study, the medical records of patients with type 2 diabetes treated at three diabetes outpatient centers of the national health system during 2006 and 2012 were examined. Only patients with at least six months of follow-up prior to the recorded examination were included. The prescription cost was calculated in Euros per patient-year (€PY).

**Results:**

A total of 1953 medical records (938 from 2006 and 1015 from 2012) were included. There were no significant differences in adjusted HbA1c, systolic blood pressure and HDL-C, while significant reductions were observed in LDL-C and triglycerides. In 2012, a higher proportion of patients were prescribed glucose-lowering, lipid-lowering and antihypertensive medications. Almost 4 out of 10 patients were prescribed the new incretin-based medications, while the use of older drugs, except for metformin, decreased. A significant increase in the adjusted glucose-lowering prescription cost (612.4 [586.5-638.2] €PY vs 390.7 [363.5-418.0]; p < 0.001) and total prescription cost (1306.7 [1264.6-1348.7] €PY vs 1122.3[1078.1-1166.5]; p < 0.001) was observed. The cost of antihypertensive prescriptions declined, while no difference was observed for lipid-lowering and antiplatelet agents.

**Conclusions:**

During the economic crisis, the cardiovascular risk indices of Greek patients with type 2 diabetes being followed in public outpatient diabetes clinics did not deteriorate and in the case of lipid profile improved. However, the total prescription cost increased, mainly due to the higher cost of glucose-lowering prescriptions.

## Background

Type 2 diabetes (T2D) is increasingly becoming more prevalent throughout the world [1]. Nowadays, multifactorial medical management of the disease is widely recommended, in order to prevent its catastrophic complications [2]. Multifactorial management includes intensive treatment and implementation of strict targets for cardiovascular risk factors (CRF), namely, hyperglycemia, hypertension, dyslipidemia and prothrombotic state [2].

We had previously reported that in Greek patients with T2D, being regularly examined in diabetes outpatient clinics of the Greek National Health System (GNHS), the management of major modifiable CRF improved significantly between 1998 and 2006. This improvement was associated with a 2.2 fold increase in the cost of the relevant prescribed medications [3]. Although the increased prescription cost could be partly attributed to the intensification of therapy, it was, however, most likely driven by the introduction of new and more expensive medications [3]. Similar trends have been observed in other countries as well [4-6].

Since our previous report, a new class of glucose-lowering medications has emerged, based on the incretin phenomenon. Medications of this class, categorized as dipeptidyl peptidase-IV inhibitors (DPP-IVi) and glucagon-like peptide-1 receptor agonists (GLP-1RA), have been widely prescribed in patients with T2D worldwide. In the year 2009, a major economic crisis emerged in Greece, leading to the implementation of a financial rescue plan lead by the “Troika”, (the International Monetary Fund, the European Commission, and the European Central Bank), in collaboration with the Greek Government. The “control” of GNHS-related expenses held a central role of the plan, which included a radical health system reform. There have been “signs” that health outcomes have worsened since the crisis emerged, especially in vulnerable groups [7], while one study reported a significant increase in the prevalence of suicidal ideation and suicide attempts [8]. Soon after the crisis explosion, negotiations between the Greek government and the pharmaceutical companies lead to consecutive reductions in virtually all medication prices, reaching an average of 25-30%, compared to the before-crisis costs. In addition, a generic-oriented drug prescription policy has been promoted [9].

The aim of the present study is to compare the clinical indices (glucose control, blood pressure and lipid profile) indicating the level of CRF control, before (2006) and during the Greek economic crisis (2012), as well as the prescription cost of medications aiming at these factors, during the same period.

## Methods

Three major GNHS diabetes outpatient clinics, one located in Athens and two in Piraeus participated in the study. A Diabetes Center in Greece is defined as an outpatient clinic running within general hospitals of the GNHS, equipped with health professionals specialized in the field of diabetes.

We compared the medical records of all patients who were examined at the above centers during 2006, with those who were examined at the same centers during the first nine months of 2012. Only patients with at least six months of follow-up by the medical staff of the same center prior to the recorded visit and patients who had at least one previous examination during this time period were included. When more than one patient visit (fulfilling the above criteria) was recorded during the same year, the first one was included.

Patients with type 1 diabetes were excluded from the study, based on indications existing on each patient’s file. In addition, patients with “unknown” type of diabetes or with indications such as “under investigation”, “sterinoid” and “LADA” were excluded as well.

According to routine clinical practice in all the participating centers, an HbA1c measurement is performed at every scheduled examination (usually every 3–4 months), both in 2006 and 2012. In addition, a complete lipid profile is routinely scheduled every six months. If an HbA1c measurement and/or complete lipid profile (total cholesterol, LDL, HDL and triglycerides) was missing, the next examination of the patient was recorded. If no such examination existed within the year under study, the patient was excluded.

Prescriptions were identified on the basis of physician’s reporting on each medical record at the recorded visit. The cost of medications was calculated in Euros per patient per year (€PY). For both periods, the official Greek market prices (as indicated on the medication’s market box) were used. Due to the fact that, starting from 2009, consecutive reductions in the prices of virtually all medications have been applied, no correction for inflation was needed.

The study protocol was approved by the Ethics Committee of all three participating institutions (Ethics Committee at Laiko General Hospital, Ethics Committee at Tzanio Hospital and Ethics Committee at Nikaia General Hospital).

### Statistical analysis

Analysis of the data was performed using the SPSS statistical software package (IBM-SPSS 20.0). Categorical data were compared using the chi-square test. Comparisons of normally distributed data between groups were performed by the independent samples Student’s *t*-test or by ANOVA. Analysis of covariance (ANCOVA) was used in order to adjust the compared means for confounders. Regarding non-normally distributed data, the Mann Whitney *U* test (two independent-samples) or the Kruskall-Wallis H test was performed. P values (two-tailed) < 0.05 were considered statistically significant. All comparisons between 2012 and 2006 were performed after adjustment for age and duration of T2D.

## Results

Out of the initial review of 4124 patient records, 1953 were eligible for inclusion in the study (938 from 2006 and 1015 from 2012). Out of the 2171 excluded patients, 710 had type 1 diabetes, 654 had other types of diabetes or prediabetes and 807 did not meet the follow-up inclusion criteria. The main demographic characteristics of the participants are shown in Table 1. The mean age, the median diabetes duration and the median follow-up period at the diabetes center were all significantly higher in the 2012 group compared to the 2006 group (Table 1). The body mass index (BMI), adjusted for age and duration of diabetes, was significantly higher in 2012 as well.

**Table 1:** Main demographic characteristics of the study participants

	**2006**	**2012**	**p**
N of participants	938	1015	
Gender (males, %)	483 (51.5)	549 (54.1)	0.3
Age (years)	64.5 [63.8-65.2]	67.3 [66.8-68.0]	< 0.001
Duration of diabetes (years)*	9 [4-16]	12 [7-19]	< 0.001
Duration of follow-up (months)*	27.0 [17–38]	40.0 [18–57]	< 0.001
BMI (Kg/m^2^)^¥^	29.9 [29.5-30.3]	30.6 [30.2-31.0]	0.02

Data are shown as n (%) or mean value [95% CI]. *Median value [interquartile range]. ^¥^Adjusted for age and duration of diabetes.

### CRF control

After adjustment for age and duration of diabetes, glycaemic control (HbA1c level) and systolic blood pressure (SBP) did not differ between 2006 and 2012 (Table 2). On the other hand, lipid profile improved significantly, in terms of total cholesterol, LDL-cholesterol (LDL-C) and triglyceride levels, while HDL-cholesterol (HDL-C) levels were similar. There was a small but statistically significant decrease in diastolic blood pressure (DBP) (Table 2). The proportion of patients achieving the target of HbA_1c_ <7% (53 mmol/mol) was 53.9% in 2012 versus 56.1% in 2006 (p = 0.3). The proportion of patients achieving the SBP target of < 140 mmHg was 59.8% and 59.7% respectively (p = 0.9), while the target of LDL-C < 2.6 mmol/l was achieved by 60.4% of patients in 2012, compared to 40.2% in 2006 (p < 0.001).

**Table 2:** Glycaemic, lipid and blood pressure profile in 2006 and 2012 after adjustment for age and duration of diabetes

	**2006**	**2012**	**p**
HbA_1_c (%)	7.0 [6.9-7.1]	7.0 [7.0-7.1]	0.7
HbA_1_c (mmol/mol)	53 [52–54]	53 [53–54]	
Non-insulin treated	6.7 [6.6-6.7]	6.7 [6.6-6.8]	0.9
	50 [49–50]	50 [49–51]	
Insulin treated	7.6 [7.5-7.8]	7.6 [7.4-7.7]	0.5
	60 [58–62]	60 [57–61]	
SBP (mmHg)	135.4 [134.2-136.7]	134.4 [133.3-135.5]	0.2
DBP (mmHg)	77.2 [76.6-77.9]	76.2 [75.5-76.8]	0.03
TC (mmol/l)	4.9 [4.8-5.0]	4.4 [4.3-4.4]	< 0.001
HDL-C (mmol/l)	1.3 [1.2-1.3]	1.2 [1.2-1.3]	0.3
LDL-C (mmol/l)	2.9 [2.9-3.0]	2.5 [2.5-2.6]	< 0.001
TGL (mmol/l)	1.7 [1.6-1.7]	1.6 [1.5-1.6]	0.003

(Data are shown as mean value [95% CI]).

SBP: Systolic blood pressure, DBP: Diastolic blood pressure, TC: Total cholesterol, TGL: Triglycerides.

### Pattern of drug treatment

Although the proportion of patients using non-insulin vs. insulin-based treatments did not differ significantly (after adjustment for age and diabetes duration), the choice between the available glucose-lowering drug classes changed (Table 3). The use of metformin increased (80.4%, compared to 66.8%), while the use of sulphonylureas decreased, from 44.3% in 2006 to 26.6% in 2012 (Table 3). The use of TZDs and glinides decreased from 17% and 12.5% in 2006 to 4.9% and 2.7% respectively in 2012. Incretin -based therapies (not available in Greece during 2006) were prescribed to 38.5% of patients in 2012. Regarding the use of insulin, a significant increase in the proportion of patients treated with ≥ 3 injections was observed in 2012. In addition, patients treated with insulin analogs increased significantly, reaching 72.9% of insulin-treated patients in 2012, compared to 49.8% in 2006 (Table 3).

**Table 3:** Pattern of antidiabetic drug treatment in 2006 and 2012

	**2006**	**2012**	P*
**Lifestyle intervention only**	38 (4.1)	11 (1.1)	0.001
**OAD only**	597 (63.6)	573 (56.5)	0.7
OAD Monotherapy^**^	165 (27.6)	182 (31.8)	0.4
Combination (2 drugs)^**^	307 (51.5)	245 (42.7)	
Combination (3 drugs)^**^	125 (20.9)	146 (25.5)	
Metformin	627 (66.8)	816 (80.4)	< 0.001
Sulphonylurea	416 (44.3)	270 (26.6)	< 0.001
TZD	159 (17.0)	50 (4.9)	< 0.001
Glinide	118 (12.5)	27 (2.7)	< 0.001
Acarbose	73 (7.8)	22 (2.2)	< 0.001
DPP-IV inhibitor	0	350 (34.5)	
GLP-1 agonist	0	39 (3.8)	
**Insulin**	303 (32.3)	432 (42.6)	0.09
1 injection^#^	91 (30.0)	126 (29.2)	0.001
2 injections^#^	118 (38.9)	83 (19.3)	
≥ 3 Injections^#^	94 (31.1)	222 (51.5)	
Insulin analog^#^	151 (49.8)	315 (72.9)	<0.001
Units per Kg of body weight	0.5 (0.3)	0.5 (0.3)	0.87

Data are shown as n (%).

*Statistical significance was assessed after adjustment for age and duration of diabetes.

**Percentage refers to those treated with OAD only.

^#^Percentage refers to those treated with insulin.

OAD: Oral antidiabetic drugs, TZD: thiazolidinediones, GLP-1: glucagon-like peptide-1, DPP-IV: dipeptidyl peptidase-IV.

The proportion of patients using antihypertensive drugs increased from 74.7% in 2006 to 84.4% in 2012 (p = 0.03). (Table 4). Among those treated for hypertension, the proportion of patients using ≥ 2 antihypertensive class medications increased from 27.7% to 39.2% (p < 0.001). The adjusted mean number of prescribed antihypertensive drug classes per patient increased from 1.5 to 1.8 (p < 0.001).

**Table 4:** Prescription cost per patient-year for all diabetes-related medications (€PY)

	**2006**	**2012**	**p**
Glucose-lowering (all patients)	390.7 [363.5-418.0]	612.4 [586.5-638.2]	< 0.001
Glucose-lowering (only treated patients)	405.3 [377.6-433.1]	621.1 [595.1-647.1]	< 0.001
OAD only	324.9 [297.0-352.9]	504.5 [476.3-532.7]	< 0.001
Insulin	553.8 [501.6-606.0]	763.8 [720.6-807.0]	< 0.001
Excluding incretins	405.3 [501.6-606.0]	508.0 [474.7-541.3]	< 0.001
Excluding incretins & adjusting for insulin analog use	442.4 [415.2-469.6]	463.2 [431.5-495.0]	0.34
Antihypertensive (all patients)	285.3 [270.6-300.1]	255.2 [241.1-269.2]	0.004
Antihypertensive (only treated patients)	371.5 [356.0-387.1]	318.7 [304.7-332.7]	< 0.001
Lipid-lowering (all patients)	357.3 [335.0-379.5]	361.0 [339.9-382.1]	0.81
Lipid-lowering (only treated patients)	571.8 [547.2-596.4]	449.9 [429.5-450.3]	< 0.001
Antiplatelet (all patients)	89.0 [75.8-102.1]	78.1 [65.6-90.7]	0.25
Antiplatelet (only treated patients)	169.8 [146.2-193.4]	146.4 [124.2-168.5]	0.16
Total	1122.3 [1078.1-1166.5]	1306.7 [1264.6-1348.7]	< 0.001

Data are shown as mean [95% CI]. Treated patients are considered as those receiving the respective class of medication. All comparisons have been adjusted for age and duration of diabetes.

The use of lipid-lowering medications increased from 61.2% in 2006 to 79.9% in 2012 (p < 0.001). The use of statins increased from 56.2% to 75.8% (p < 0.001), while the use of fibrates did not change (3.4% in 2006 and 2.1% in 2012, p = 0.3). A significant increase in the use of ω-3 fatty acids and ezetimibe was observed (10.7% vs. 1.6% and 11.6% vs. 5.9%, both p < 0.001). Combining statins with any other lipid-lowering medication increased from 9.4% in 2006 to 25.9% in 2012 (P < 0.001).

The proportion of patients being prescribed antiplatelet medications did not change between 2006 and 2012 (52.2% vs. 51.1% respectively, p = 0.6). However, the pattern of treatment changed substantially, showing a decline in aspirin use (37.0% vs. 43.6%, p = 0.002) and an increase in clopidogrel use (18.6% vs. 10.8%, p < 0.001). The use of dual antiplatelet therapy increased by borderline significance (4.8% vs. 3.1%, p = 0.06).

### Cost of treatment

As shown in Table 4, a significant increase in the total prescription cost per patient-year was observed in 2012. The mean total cost of all diabetes-related medications increased by 20.9% (1324.1 ± 729.2 €PY vs. 1096. ± 634.0 €PY, p < 0.001). After adjustment for age and duration of diabetes the difference declined to +16.4% (1306.7 €PY vs. 1122.3 €PY, p < 0.001). A significant increase was observed for glucose-lowering medications (56.7%), while a non-significant 1.0% increase was found for lipid-lowering medications. On the other hand, a significant 10.6% decline in the cost of antihypertensive drugs and a non-significant 12.2% decline in the cost of antiplatelet drugs was observed.

**Figure 1:**
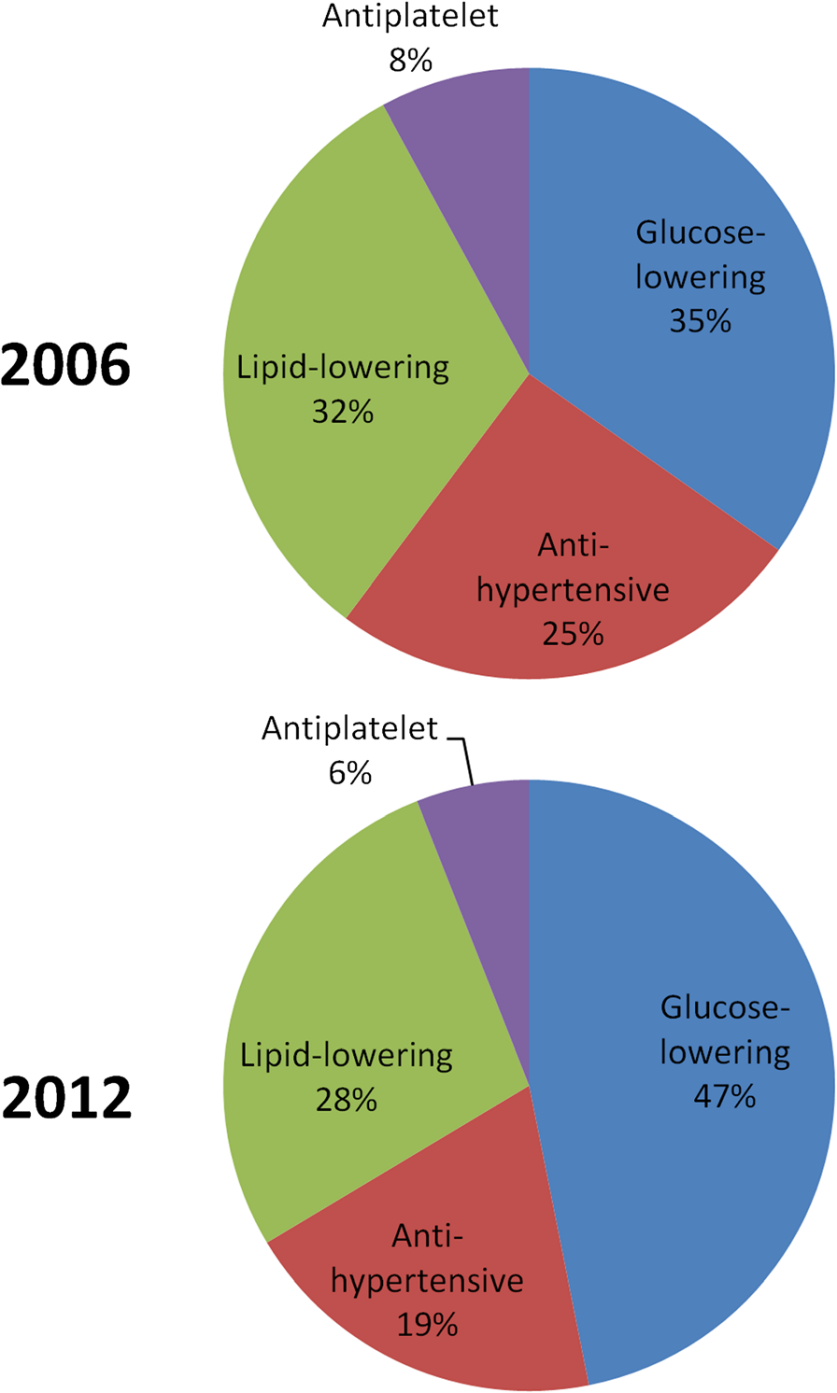
Relative contribution on prescription cost of each class of medications used for the management of cardiovascular risk factors.

The 2012 sub-group being treated with incretin-based therapies (n = 389) had a 2.5-fold higher adjusted glucose-lowering prescription cost, compared to patients using older therapies (n = 504), after excluding insulin-only treated patients (944.5 [903.8-985.2] €PY vs. 374.4 [338.6-410.2] €PY), while their adjusted HbA1c was slightly higher (7.1 [7.0-7.2] vs. 7.0 [6.9-7.1], p = 0.13).The relative contribution of glucose-lowering medications to the total prescription cost increased from 34.8% in 2006 to 46.9% in 2012, while all the other diabetes-related prescribed medications showed a decline in their relative contribution (Figure 1).

## Discussion

The principle findings of this study are: 1) There is no deterioration of clinical indices related to cardiovascular risk factors of patients with T2D who are regularly visiting public outpatient diabetes clinics, after the emergence of a major economic crisis in Greece. 2) The cost of medications prescribed for cardiovascular risk management of these patients increased by 15% in 2012 (during the crisis), compared to 2006 (before the crisis).

We had previously reported that glycaemic control of patients with T2D, who were examined in diabetes outpatient clinics, improved by approximately one HbA1c % unit between the years 1998 and 2006 [3]. The HbA1c improvement had been attributed to several factors, but perhaps, most importantly, to the publication of the UKPDS trial results, showing that intensive treatment of hyperglycemia has beneficial effect on diabetes complications [10]. Moreover, in 2006 we had observed a modest reduction in blood pressure and a striking reduction in LDL-C of T2D patients, due to a more intensified antihypertensive therapy and to the widespread use of statins. These improvements can be attributed to the wide implementation of multifactorial management, which had been shown to decrease substantially both morbidity and mortality, associated with T2D [11].

In the present study, as opposed to our previous one, no further decline in the mean adjusted HbA1c and SBP values have been observed. The proportion of patients achieving HbA1c <7% (53 mmol/mol) has remained stable at around 50%, as well as that of patients achieving SBP < 140 mmHG, at around 60%. In the USA National Health and Nutrition Examination Survey (NHANES) cohort, there was no further improvement in glycaemic control between 2003/2006 and 2007/2010, while, similarly to our results, a significant increase in the proportion of patients achieving an HbA1c < 7% (53 mmol/mol) was observed between 1999/2000 and 2003/2006 [12]. This might be due to several factors. The enthusiasm produced after the publication of the UKPDS results in 1998 in contrast to the skepticism, generated from the ACCORD and VADT trials in 2009, might be an explanation. On the other hand, in the case of Greece, the economic crisis might have contributed as well. It should be emphasized, however, thet the LDL-C target of <100 mg/dl (2.6 mmol/l) was achieved, by 60% of patients in 2012, compared to only 40% in 2006, a finding that can be clearly attributed to the continuing spread of statin use (Table 3).

It has been speculated that the economic crisis might deteriorate health status of Greek citizens due to austerity and deprivation of health care resources [9,13]. Due to the financial crisis, the Greek government negotiated with pharmaceutical companies and decided a “haircut” for most licensed medications in the Greek market that reached an average of 30% compared to pre-crisis [9]. Importantly, during the crisis, the GNHS continued to reimburse 90% of prescription cost regarding all licensed medications for T2D, including incretin-based therapies and insulin analogs.

In Table 5, the prices before and after the crisis explosion are shown for some key medications, commonly used for the treatment of T2D [14]. Despite the price reduction, however, according to our results, the total prescription cost for T2D increased significantly in 2012. As clearly shown in Table 4, the prescription cost related to lipid-lowering, and antiplatelet medications did not virtually change, despite that a higher proportion of patients were prescribed lipid-lowering medications. Furthermore, the prescription cost of antihypertensive medications decreased significantly despite intensification of antihypertensive therapy (Table 4). On the contrary, the adjusted prescription cost of glucose-lowering medications increased by 56.7% (Table 4). Taking into account that the price of all the older glucose-lowering medications decreased in 2012 (Table 5), it can be assumed that the prescription cost rise is (at least partly) drawn by the introduction of new and more expensive medications. Indeed, when patients taking DPP-IV inhibitors and GLP-1 agonists were excluded (Table 4), the glucose-lowering prescription cost difference between 2006 and 2012 dropped to +25.3%, still highly significant. However, when further adjusting for the use of insulin analogs (Table 4), the difference between 2006 and 2012 almost disappeared and became non-significant (+4.7%, p = 0.34).

**Table 5:** Comparison of medication prices between 2007 (before the economic crisis) and 2012 (during the crisis) for key diabetes-related medications

	**2007**	**2012**	**% reduction**
Metformin 1700 mg	4.6	4.2	-9.5
Glimepiride 3 mg	15.0	9.8	-34.7
Glimepiride 3 mg [generic]	12.0	8.8	-26.7
Pioglitazone 30 mg	65.2	45.1	-30.8
Insulin Glargine 1500 i.u.*	84.5	63.7	-24.6
Insulin Premixed 30/70 (Human)**	33.7	30.8	-8.6
Ramipril 5 mg	18.9	14.5	-23.3
Indapamide 1.5 mg	9.2	7.4	-19.6
Amlodipine 10 mg [generic]	22.8	11.3	-50.4
Simvastatin 40 mg	55.9	35.9	-35.8
Atorvastatin 20 mg [generic]	41.7	20.4	-51.1
Aspirin 100 mg	1.2	1.5	+25
Clopidogrel 75 mg	67.9	32.5	-52.1

Price is referred to € per 30-day treatment.

*Prefilled pen. **Cartridges.

Insulin analogs have been associated with a lower risk of hypoglycemia than older human insulins [15,16]. Since the mid 00’s, several compounds based on the incretin phenomenon have been added in the armamentarium of glucose-lowering medications. Two GLP-1 analogs (exenatide and liraglutide) and three DPP-IV inhibitors (sitagliptin, vildagliptin and saxagliptin) have been introduced in the global and the Greek market as new glucose-lowering agents. Although in the 2009 American Diabetes Association/European Association for the Study of Diabetes (ADA/EASD) consensus statement on management of hyperglycemia, only exenatide was included as second or third line option treatment for T2D [17], the subsequent 2012 ADA/EASD position statement has assigned to all incretin-based therapies a central role in T2D management [18]. In many countries nowadays, GLP-1 based therapies hold a considerable share in diabetes-related prescriptions [19,20]. The main advantage of incretin-based medications is a neutral (with DPP-IV inhibitors) or favorable (with GLP-1 agonists) effect on body weight and the lack of significant risk for hypoglycemia. On the other hand, incretin-based therapies do not seem to be more potent than previously existing medications in terms of HbA1c reduction, while there is some concern regarding their possible association with acute and/or chronic pancreatitis and α-cell hyperplasia [21-23]. In our 2012 group the HbA1c of patients receiving incretin-based therapies did not differ from those taking older drugs. Unfortunately, we were not able to estimate hypoglycemic episodes, since they were not reliably recorded. The BMI of patients receiving incretin-based drugs was actually higher, compared to those under older oral antidiabetic agents, but this can be attributed to indication bias.

Cost-utility analysis in the UKPDS study [24] showed that tight vs. less tight blood pressure (BP) control, as well as intensive vs. conventional glucose control, was highly cost-effective. A cost-effectiveness study based on the results of the Steno-2 trial demonstrated that in patients with T2D at high risk for CVD, multifactorial intervention aiming at strict control of major risk factors for CVD can be cost-effective, especially if administered in a primary care setting [25].

Cost-effectiveness of the new therapies is under investigation [26,27]. It has been repeatedly reported that the largest components of medical expenditures attributed to diabetes are associated with treatment of complications (hospital inpatient care and medications to treat these complications) rather than direct prescription cost to treat hyperglycemia [28,29]. Hence it can be argued that the benefit derived from reduced incidence of hypoglycemia (including severe hypoglycemia) and reduction (or at least not increase) of body weight counterbalance the higher medication price. Prospective studies with hard diabetes-related endpoints and independent cost-effectiveness studies of high quality are needed to establish cost-utility of new therapies. Our study, due to its retrospective design, cannot be used to draw conclusions concerning cost-effectiveness calculations. In any case, current guidelines issued by the ADA/EASD and the American Association of Clinical Endocrinologists encourage the use of GLP-1 based therapies as second-line options [18,30].

The main limitation, of the present analysis is the questionable representativeness of the study population. A diabetes center in Greece is defined as an outpatient diabetes clinic running within a public or university general hospital, equipped with health professionals specialized in the field of diabetes. Any person regardless of having a public insurance or not, may have an appointment and examination at these centers, with a wait time of 2-4 months. For those not being insured, there is a 5€ charge that includes full medical examination and one finger-stick glucose measurement. Due to the lack of an organized primary care system in Greece, many patients use the hospital outpatient clinics as a substitute for primary care management, even for reasons of drug prescription. The patient population consists mainly of lower socio-economic status and unemployed individuals, as well as pensioners, while employers are a minority, mainly because the outpatient clinics function only during the morning. We cannot exclude, however, the possibility that several patients, especially in the midst of a major economic crisis, may not afford a 5€ fee. Furthermore, unemployed/uninsured patients may not visit the diabetes clinics, due to their inability to meet financial obligations associated with medical advises/prescriptions. Costs associated with transportation might be a barrier as well. In addition, patients followed by general practitioners or in the private setting are not included in this analysis. In summary, although not representative of the whole Greek T2D population, the study participants cover a broad range of middle to low socioeconomic class patients.

## Conclusions

In conclusion, we observed that during the Greek economic crisis, cardiovascular risk management indices of T2D patients being examined at three major diabetes outpatient clinics of the GNHS, did not deteriorate and in the case of LDL-C and triglycerides, they improved. A 15% increase in the associated total prescription cost was observed, mainly due to the higher cost of glucose-lowering related medications. The introduction of new medications in this field (as opposite to the unchanged armamentum against hypertension, dyslipidemia and hyperthrombotic state), although responsible for the increased direct cost, might, on the other hand, provide long-term benefits associated with a favorable profile in terms of hypoglycemic risk and body weight. Long-term prospective studies with hard diabetes-related endpoints and independent cost-effectiveness studies of high quality are needed in order to clarify cost-effectiveness of new therapies.

## Abbreviations

€PY: Euros per patient-year; ADA: American Diabetes Association; BMI: Body mass index; CRF: Cardiovascular risk factor; CVD: CardioVascular disease; DPP-IV: Di-Peptidyl peptidase-IV; EASD: European Association for the Study of Diabetes; GLP-1: Glycogen like peptide-1; GNHS: Greek national health system; LADA: Latent autoimmune diabetes of the adults; OAD: Oral antidiabetic drugs; T2D: Type 2 diabetes.

## Competing interest

Dr. LIATIS reports grants, personal fees and non-financial support from NOVO NORDISK, grants, personal fees and non-financial support from SANOFI, grants, personal fees and non-financial support from NOVARTIS, grants and personal fees from MSD MERK, grants and non-financial support from PHARMASERV LILLY, outside the submitted work.

Drs Papaoikonomou, Ganotopoulou, Papazafiropoulou, Dinos, Michail and Xilomenos have nothing to disclose.

Dr. MELIDONIS reports personal fees from NOVO NORDISK, personal fees from SANOFI, personal fees and non-financial support from NOVARTIS, personal fees and non-financial support from PHARMASERV LILLY, outside the submitted work.

Dr. PAPPAS reports grants and non-financial support from SANOFI, grants and non-financial support from NOVARTIS, grants and personal fees from MSD MERCK, grants and personal fees from PHARMASERV LILLY, grants and personal fees from ASTRA ZENECA, grants and personal fees from BIANEX, outside the submitted work.

## Authors’ contribution

Dr SL contributed to the conception, design, statistical analysis and writing of this manuscript. Dr SP contributed to the design, data collection and data analysis. Drs AP, AG and CD contributed to data collection and data analysis. Drs MM and AX contributed to data collection and writing of the manuscript. Dr AM contributed to the design of the study and data interpretation. He also revised the manuscript. Dr SP contributed to the design of the study and data interpretation. He also revised the manuscript. All authors read and approved the final manuscript.

## Pre-publication history

The pre-publication history for this paper can be accessed here:

http://www.biomedcentral.com/1472-6823/14/23/prepub
